# Efficacy of a Standardised Patient Simulation Programme for Chronicity and End-of-Life Care Training in Undergraduate Nursing Students

**DOI:** 10.3390/ijerph182111673

**Published:** 2021-11-06

**Authors:** Silvia Escribano, María José Cabañero-Martínez, Manuel Fernández-Alcántara, Sofía García-Sanjuán, Rafael Montoya-Juárez, Rocío Juliá-Sanchis

**Affiliations:** 1 Department of Nursing, Faculty of Health Sciences, Institute for Health and Biomedical Research (ISABIAL), University of Alicante, 03690 San Vicente del Raspeig, Spain; silvia.escribano@ua.es (S.E.); sofia.garcia@ua.es (S.G.-S.); rjulia@ua.es (R.J.-S.); 2 Department of Health Psychology, Faculty of Health Sciences, University of Alicante, 03690 San Vicente del Raspeig, Spain; mfernandeza@ua.es; 3 Department of Nursing, Faculty of Health Sciences, University of Granada, 18016 Granada, Spain; rmontoya@ugr.es

**Keywords:** efficacy, nursing, undergraduate, standardised patient, simulation with a standardised patient, palliative care, gerontology

## Abstract

Background: Standardised patient simulations seem to be useful for improving the communication skills of health sciences students. However, it is important to define the effectiveness of these types of interventions in complex scenarios linked to disease chronicity and end-of-life contexts. Methods: A quasi-experimental study with pre- and post-intervention measures was carried out in a single group. A total of 161 nursing students completed different assessment instruments to measure their attitudes towards communication (Attitude Toward Communication Scale), self-efficacy (Self-Efficacy of Communication Skills, SE-12), and communication skills (Health Professionals Communication Skills Scale, EHC-PS) before and after simulation training with standardised patients. The objective of the program was to train students in non-technical skills for complex situations involving chronicity and end-of-life care. It comprised eight sessions lasting 2.5 h each. Results: The results showed notable baseline gender differences in attitudes towards communication and in the informative communication dimension, with women obtaining higher scores. The participants’ self-efficacy and communication skills significantly improved after completing the intervention, with no significant differences being found for the attitudes towards communication variable. Conclusion: The standardised patient simulation programme for complex scenarios related to chronicity and end-of-life contexts improved communication self-efficacy and communication skills in these nursing students. In future work it will be important to analyse the influence of gender and attitudes towards communication as variables in the learning of communication skills in nursing students.

## 1. Introduction

The European framework for higher education supports the promotion of lifelong learning, knowledge acquisition, and the assessment of professional competencies [1], understanding them as the degree to which students use their knowledge, attitudes, and judgment associated with their profession to resolve arising situations [2]. One of the most used and successful methods to facilitate learning and the development of competencies in health sciences students is simulation-based education (SBE) [3]. SBE allows students to identify aptitudes that they must improve and offers them an opportunity to repeatedly practice and develop their skills in a safe environment. This reduced the occurrence of adverse events secondary to high levels of stress or anxiety in nursing students [4,5].

Over the last decade, SBE has been implemented to help develop communication skills in clinical settings [6]. Communication skills are part of the patient–health professional interpersonal relationship and directly affect the therapeutic process [7]; they are a core element in the provision of support and care in palliative care as well as for other complex patients [8]. In the context of communication skills training, perceived self-efficacy and student attitudes towards these skills are two variables of great importance. Self-efficacy refers to a student’s perceived ability to communicate [9] and is an important predictor of their motivation, learning, and performance [10]. Positive attitudes towards communication are associated with a higher level of satisfaction and an improved quality of delivered care by students [11].

Therefore, students might receive specific training in emotionally complex situations [12] prior to their incorporation into professional teams [13]. Inadequate training in palliative and chronic care could lead to greater emotional distancing, difficulty in decision making, and avoidance behaviours both in these situations as well as with patients and their families [14].

In this sense, the use of standardised patients (SPs) has considerably increased in communication skills training programmes for health sciences students, especially in the areas of oncology and palliative care [15,16]. SPs provide students with realistic interactions and responses to controlled and evaluable situations through protocolised simulations and the use of validated assessment methods [17]. In addition, studies have shown that the use of trained actors confers greater realism and fidelity to the scenarios presented in communication skills training programs [18], especially in the context of emotionally complex situations [19]. Some authors have studied how participation in end-of-life care simulation programs involving SPs endows trainees with more positive attitudes towards the care of dying patients and significantly improves their self-efficacy [20,21].

These results have also been reflected in studies in other subject areas. For example, a study with a quasi-experimental design in the field of mental health showed that simulations improve students’ attitudes and enhanced their confidence and self-efficacy [22]. Other quasi-experimental design studies in the field of emergency medicine found significant and favourable relationships in the dimensions of satisfaction, confidence, and motivation following the performance of high-fidelity simulations by students [23,24]. Therefore, the benefits of high-fidelity simulations in terms of student learning calls for further research in this field [25,26].

The current academic literature reflects a growing interest in communication skills training through simulations with SPs [27,28,29,30,31]. However, the variability in the methodologies used (simulation with actors, trained professionals, students, or a combination of all the above), the different designs (quasi-experimental, exploratory, or mixed studies), and the variability in the sample sizes (with a range between *n* = 12 and *n* = 630) [16,17] highlight the lack of protocolised structures and properly validated tools, [32]. Thus, it is important to evaluate the effectiveness of these programme types by applying quantitative methodologies and by using tools to obtain objective, valid, and reliable information [16]. This will allow for the adequate assessment of student attitudes towards the communication skills set out in their training curriculum [33].

Therefore, there is growing interest in exploring specific simulation scenarios with SPs in end-of-life care education in nursing students, especially focusing on SP simulation programmes. Thus, the object of this current research study was to evaluate the effectiveness of a simulation programme using SPs in complex scenarios linked to chronic and end-of-life situations in terms of the variables of attitude, self-efficacy, and communication skills among nursing students.

## 2. Materials and Methods

### 2.1. Design and Participants

A quasi-experimental study was carried out in a single group with pre- and post-intervention measurements. To clearly report the elements of the study we followed the extended guidelines of the Consolidated Standards of Reporting Trials (CONSORT) for simulation-based reports [34]. All eligible participants (*n* = 204) who completed the simulation were fourth-year students in the nursing undergraduate course during the academic period of 2019/2020 at the University of Alicante. The groups were formed according to the enrolment of fourth-year nursing students in one of the 12 laboratory practice groups (A–L) of each theoretical subject in the first quarter.

Each group (comprising 17–18 students) freely divided themselves up into a total of 6 subgroups, each with 2–3 members. Therefore, a total of 72 subgroups were created in order to implement the SP intervention. These subgroups remained unchanged throughout the training except in 2 cases, because of abandonment of the programme by one participant in each subgroup, leaving 2 members in each subgroup in these cases. The natural opportunity presented by the enrolment of nursing students into this course was used to obtain a convenient sample.

### 2.2. Intervention Programme: A High-Fidelity Simulation

A simulation programme with SPs was implemented between September and December 2019. The home care simulation classrooms in the Faculty of Health Sciences of the University of Alicante were used. As described in Table 1, the objective of the programme was to train the students in non-technical skills (communication) for complex care situations in the context of chronicity and end-of-life care [18,19]. The teaching team that implemented the programme designed 12 cases with contents related to grief (*n* = 3), decision making and coping in difficult situations (*n* = 3), states of acute confusion in the context of chronicity (*n* = 2), bad news (*n* = 1), the pact of silence (*n* = 1), pain management (*n* = 1), and contexts in the last days of life (*n* = 1) [12,15,19,20,21].

The programme comprised 8 sessions, each lasting 2.5 h (Table 1). The first two sessions were dedicated to preparing the students prior to the simulation scenario training. The objectives of these initial sessions were for the students to understand the educational intervention and cases that would be presented and to generate a known and safe environment for the groups by promoting group dynamics. Two cases were randomly assigned to each subgroup (i.e., 6 per training group), with the aim of developing an adequate theoretical appreciation of the intervention types. Two weeks before the start of the training, the students shared their understanding of the cases they had been assigned with the rest of their group. The remaining 6 sessions were specific training sessions and followed a standardised structure which had been agreed upon by the research team (Table 2). Each subgroup actively participated in 4 different simulation scenarios, while they participated in the rest of the scenarios (*n* = 20) both passively as observers and actively during the debriefing. An absence rate of up to 20% was permitted.

Three professional actors who were experts in improvisation participated as the SPs. Prior to completing the scenario, all the actors were given a description of each case and both the objectives of the interaction with the students and the appropriate interventions for each of the simulations were explained to them. In addition, all the programme teachers involved received specific training about the interventions (lasting 4 h) which addressed content related to group management, the structure of the simulation sessions, protocols to follow, and the management of problem situations during the simulation and debriefing sessions. The session structure for each scenario was: pre-debriefing, presentation of the case by the students, simulation, and a structured group debriefing during the simulation session, as suggested in the literature [35,36].

### 2.3. Variables and Instruments

A data collection form was built which included the following questionnaires:

A questionnaire about sociodemographic characteristics related to communication skills, which included the variables of gender, age, nationality, and training in communication skills through two questions: “Have you received training in social/communication skills during your nursing degree training?” and “Have you received training outside your nursing degree in social/communication skills?”.

The attitudes towards communication were evaluated using the Spanish version (*Attitudes Towards Health Communication* [37]) of the *Attitudes Towards Medical Communication Scale* [38]. This one-dimensional instrument comprises 11 assessable items measured on a 5-option Likert-type response scale from “strongly disagree” (1) to “strongly agree” (5). The overall score ranges from 11 to 55 points, with higher scores reflecting more positive attitudes towards communication. The Spanish version of the scale has an adequate internal consistency score of 0.75 [37].

Self-efficacy in communication skills was evaluated by employing the Spanish version (manuscript in preparation) of the *Self-Efficacy of Communication Skills* (SE-12) scale [39]. This one-dimensional scale contains 12 items assessed on an 11-point Likert-type response scale ranging from “very insecure” (1) to “very secure” (10). The overall SE-12 score can range from 12 to 120 points, with higher scores indicating greater confidence in one’s own communication skills. This scale has a high internal consistency (Cronbach alpha = 0.95) and adequate test–retest reliability (intraclass correlation coefficient = 0.71) in its original version [39], and has been validated in Spanish students with an internal consistency of 0.94 (manuscript in preparation).

Communication skills were evaluated through the *Health Professionals Communication Skills Scale* (EHC-PS), an 18-item instrument comprised of 4 communication skills dimensions: empathy, informative communication, respect, and social ability/assertiveness [40]. The EHC-PS is assessed on a Likert-type response scale with 6 response options ranging from “almost never” = 1, to “many times” = 6. The internal consistencies of the EHC-PS dimensions were 0.77 for empathy, 0.78 for informational communication, 0.74 for respect, and 0.65 for social ability/assertiveness. When validated in nursing students, this scale showed excellent internal consistency (0.88) and moderate convergent validity (*r* = 0.35, *p* < 0.001) with the attitudes towards communication [41].

### 2.4. Procedure

The data were collected both during the first session (September 2019) and after the implementation of the entire programme (December 2019) through an electronic Internet-based form (Google Forms) which was disseminated to the students through the institution’s internal platform known as “UACloud”. Along with the questionnaires described above, the form also included detailed information about the study, an express request for informed consent from the participants, and details about the voluntary nature of their participation in the research and about the treatment of the collected data.

To achieve a high post-intervention response rate, a standardised methodology was followed in which we sent three email reminders, one week apart each, encouraging participation and containing a link to the questionnaire. A total of 161 students completed the post-intervention questionnaire (loss rate = 21.17%).

This project was approved by the Bioethics Committee at the University of Alicante (file number UA-2018-10-24) and all participants signed their consent form to participation. All the data were treated confidentially, and participation did not impact the students’ grades. The study followed the criteria established by the Declaration of Helsinki and the Standards of Good Clinical Practice of the European Union.

### 2.5. Data Analysis

Descriptive analyses of the sociodemographic variables and the outcome variables were carried out. For the attitude, self-efficacy, and perceived communication skills variables, we first checked the assumption of normality for each one by examining the Kolmogorov–Smirnov test. Next, we checked whether there were any significant differences in the scores at the baseline by applying Student’s *t*-tests for the gender variable and Pearson tests for the age variable. Effect sizes were calculated for variables with statistically significant differences, and their threshold values were interpreted as small (0.20), moderate (0.50), and large (0.80) effects [42,43].

An analysis of variance with repeated measures (ANOVA MLG) was performed to compare the scores obtained between the pre- and post-intervention timepoints, including the sociodemographic variable of gender as a covariate when this factor had presented significant differences at baseline. Eta squared was calculated as a measure of the effect size for analysis of variance (ANOVA) models and its threshold values were interpreted as small (0.04), moderate (0.25), and large (0.64) effects [42]. All the statistical analyses were carried out using SPSS software (version 25.0; IBM Corp., Armonk, NY, USA).

## 3. Results

Of the total of 161 students who completed the questionnaire at both study timepoints, 83.2% were female (*n* = 134). The mean participant age was 22.93 years (*SD* = 5.44, range = 20–48) and 97.5% (*n* = 157) had Spanish nationality. Most of the students had received initial training in communication skills during their undergraduate nursing training (95.7%, *n* = 154), and only four (2.48%) students reported not having received any prior training in communications in any context. None of the participants had previous experience with simulation programs.

### 3.1. Communication Skills Variables

Before the simulation, our results showed moderate–high scores for the attitudes (*M* = 52.54; *SD* = 2.45), self-efficacy (*M* = 81.73; *SD* = 14.08), and communication skills (*M* = 87.41; *SD* = 8.08) perceived by the students (Table 3). At baseline, we found statistically significant differences according to participant gender for communication attitudes, whereby the female participants obtained higher scores (*M* = 52.84; *SD* = 2.20) compared to male students (*M* = 51.30; *SD* = 3.47; *t* = 2.22; and *p* = 0.03). Regarding the dimensions of the communication skills scale, statistically significant differences were found only for the informative communication dimension, in which women again achieved better scores than men (*M* = 29.77, *SD* = 2.54 vs. *M* = 28.41; *SD* = 2.89; *t* = 2.48; and *p* = 0.01). No statistically significant differences were found according to participant age or any of the variables measured at baseline. All our other results are presented in Table 3.

### 3.2. Effect of the Simulation Programme

When comparing the scores obtained at the beginning and end of the intervention programme (*n* = 161), we observed that both self-efficacy and the overall score for perceived communication skills had improved at the end of the simulation programme (*F* = 165.41; *p* < 0.001 and *F* = 50.54; *p* < 0.001, respectively), with a moderate effect size. The scores for all the dimensions of the communication skills scale improved after the intervention, except for the informational communication dimension (*F* = 1.20; *p* = 0.28). No improvement was observed for the attitudes variable (*F =* 0.67; *p* = 0.42, Table 4).

## 4. Discussion

The objective of this study was to evaluate whether a simulation programme using SPs to expose nursing students to situations involving patient chronicity and end-of-life contexts effectively improved their scores for the variables of attitude, self-efficacy, or communication skills. Our results indicate that the simulation programme proposed had improved the communication skills and self-efficacy related to communication, although there had been no improvement in the attitudes towards the communication variable.

The number of participants in our sample and the cohort composition were similar to other analogous studies. As is usual in studies on populations of nursing students, the percentage of female participants in this work was very high [44,45]. Of note, most of the students participating in this study had received prior specific training in communication skills either during their instruction in nursing or externally. This factor may have impacted their baseline communication levels in our study, resulting in moderate–elevated scores for attitude, self-efficacy, and perceived communication skills. This is unusual, given that some previous studies have shown that nursing students frequently lack communication skills when they finish their undergraduate training [46], especially those related to complex situations of chronicity or end-of-life contexts [47].

Here, we found some differences at baseline with respect to gender, with women obtaining higher attitudes towards communication scores compared to men at the beginning of the study. This effect has already been reported in previous studies, such as the work by Cleland, Foster, and Moffat [48] on medical students in Scotland, or that by Alotaibi and Alsaeedi [49] on nursing students in Saudi Arabia. Löffler-Stastka et al. [50] pointed out that women tended to have better outcomes towards communication with patients in regard to psychosocial factors, while men tended to feel more insecure in this respect. These authors concluded that these differences had been present since the time of the participants’ undergraduate training.

Regarding their perceived communication skills, the women in our study had better scores in the informational communication dimension than the men. However, there are discrepancies in the academic literature in this regard. For example, Graf et al. [51] detected differences in the communication skills of medical students based on gender, with women standing out for non-verbal language and empathy. Similar results were found in a study by Strekalova et al. [52] on nursing students from the USA, which found that women had greater skill in showing empathy than men. However, no differences were found in the communication skills of men and women in the study carried out by Shafakhah et al. [46] on nursing students from Iran. It should also be noted that many studies conducted on nursing students did not consider gender differences given the relatively low proportion of male students usually found in these populations. Therefore, future work must continue examining the influence of gender roles on communication skills in nursing.

Regarding the changes produced in this work after the implementation of the programme, our results showed that self-efficacy in relation to communication increased after completion of the simulation programme. This finding is consistent with previous studies that used similar methodologies. For example, Karlsen et al. [53] showed how nurses from Norwegian intensive care units described feeling more confident in their communication skills after participating in a simulation programme. Similarly, Kortes Miller et al. [54] evaluated the effect of a simulation using a smart mannequin with informal caregivers, and concluded that self-efficacy related to communication skills with patients at the end of their lives had improved.

It is important to remember that according to the theory of Bandura [55], self-efficacy influences the acquisition, development, and retention of new skills. People with high levels of self-efficacy tend to attain higher levels of achievements and are more persistent in reaching these goals when faced with difficulties. Therefore, achieving high levels of self-efficacy in communication could help to improve the communication skills of nursing students in the future. In this line, a study conducted with nursing students in the United States showed a significant improvement in self-reported confidence and in the usage of communication skills after a simulation-based program with SPs [56].

In another vein, and in line with Bar-Sarmiento et al. [57], the simulation programme we described in the present study also significantly improved the communication skills perceived by the nursing students in our cohort. In a study conducted on medical students in France [58], the participants also showed improved communication skills compared to those who followed the standard training programme. Some studies have suggested that communication skills should not only be assessed from the student’s own point of view, but that structured external assessments such as the *Objective Structured Clinical Examination* (OSCE) [59] should also be employed. For decades, the OSCE has been used for multiple purposes during the training of nursing students, including training in communication skills [60]. However, recent research suggests that students’ perceptions of their communication skills often tend to coincide with those of external evaluators [51,60], and so both perspectives must be considered.

Finally, no improvement was observed for the attitudes towards communication variable. These results differ with respect to what is reflected in other studies in which simulation programs improved attitudes towards communication, especially in the context of end-of-life communication [61,62]. Nevertheless, we must consider that the initial scores on this scale were already quite high relative to the maximum possible score, and so there was not much room for improvement. Other studies carried out on health sciences professionals using the same scale have reported similar scores [38]. Notwithstanding, the correlation between attitudes towards communication and the self-acquisition of skills was weak [41], meaning that this factor should be considered but should not be, a priori, a determining factor in these assessments.

Interestingly, during the COVID-19 pandemic, higher education institutions were forced to implement simulations as part of their learning methods. These included online virtual simulations of patient consultations or simulated clinical experiences, generally with satisfactory results in terms of student learning [63,64]. However, few studies used SP simulations during the pandemic, and these studies did not provide data on the effect that this intervention had on students [65]. The limitations caused by the COVID-19 pandemic meant that the implementation of simulations with SPs to help train nursing students was necessary so that students could be in contact with real patients.

In terms of the specific limitations of this work, it should be noted that, for teaching organisation reasons, the effect in the intervention group could not be compared to a control group. Thus, more experimental designs should be implemented in the future through the use of clinical trials. In addition, a notable percentage of participants (21.17%) did not complete the final questionnaire in this study. Previous work involving SPs have assumed that a maximum drop-out rate of 20% is adequate [66]. Thus, although the losses we experienced in this current work were not exceptionally high, it would nonetheless be advisable to try to improve these response rates in future research in this field. Despite this, the final sample size was quite large, exceeding that of many previous studies with standardised patients [67,68,69].

## 5. Conclusions

In conclusion, the simulation programme with SPs we implemented to address complex scenarios linked to chronic and end-of-life patient situations improved the communication self-efficacy and communication skills (empathy, respect, and assertiveness) of the nursing students included this work. The effect sizes were moderate, suggesting that the improvement was significant. In the baseline assessment, females had better scores for attitudes toward communication and in the process of informative communication compared to male students. In future work it will be important to analyse the influence of gender and attitudes towards communication as variables in the learning of communication skills in nursing students, as well as to introduce specific exercises to address and improve their attitudes toward communication and informative communication with patients.

## Figures and Tables

**Table 1:** Description of the high-fidelity simulation intervention using standardised patients implemented in this study.

**Aims**	To train effective communication skills in healthcare students.
**Target population**	Fourth-year nursing students.
**Theoretical basis**	Patient-centred care.
**Context**	Chronicity in different stages of life and end-of-life care.
**Duration of each session**	2.5 h
**Sessions**	
1. Introduction to the simulation	Description of the simulation program structure, objectives, and organisation. Establish the initial group dynamics to allow the students to get to know each other, promoting group cohesion. Organisation of the subgroups. Description and assignment of the first 6 theoretical cases.
2. Introduction to the simulation	Introduction to the intervention. Promote group dynamics and establish a spirit of teamwork. Description and assignment of the last 6 theoretical cases.
3. Simulation	Case 1: Chronic grief. Case 2: Approach to acute confusion in a chronic patient (delusions).
4. Simulation	Case 3: Perinatal grief with suicidal ideation. Case 4: Management of acute confusion in a chronic patient (agitation).
5. Simulation	Case 5: Help in coping with a complex situation. Case 6: Caregiver care.
6. Simulation	Case 7: Mourning in an old man. Case 8: Conflict in family decision making.
7. Simulation	Case 9: Pact of silence. Case 10: Bad news.
8. Simulation	Case 11: Pain management. Case 12: Addressing a near-end-of-life situation.

**Table 2:** Description of the structure of each simulation session (2.5 h).

Duration	Activity
15 min	Pre-briefing.Theoretical presentation of case 1 and feedback from course colleagues.
30 min	First simulation, case 1 (5–10 min simulation and video recording + 20 min debriefing).
30 min	Second simulation, case 1 (5–10 min simulation and video recording + 20 min debriefing).
15 min	Theoretical presentation of case 2 and feedback from course colleagues.
30 min	First simulation, case 2 (5–10 min simulation and video recording + 20 min debriefing).
30 min	Second simulation, case 2 (5–10 min simulation and video recording + 20 min debriefing).

**Table 3:** Descriptive statistics of the main variables at baseline and differences depending on sex and age (*n* = 161).

	Total	Female (*n* = 134)	Male (*n* = 27)	Student’s *t-*Test	*Df*	*p*	Effect Size ^a^	Age ^b^
Attitudes towards health communication	52.54 (2.45)	52.84 (2.20)	51.30 (3.47)	2.22	30.35	0.03	0.25	0.14
								
SE-12	81.73 (14.08)	81.07 (14.16)	84.96 (13.46)	1.44	159	0.19	-	0.05
								
EHC-PS (total scale)	87.41 (8.08)	87.82 (7.81)	85.37 (9.20)	−1.31	159	0.15	-	0.06
Empathy ^c^	25.76 (3.17)	25.94 (3.10)	24.85 (3.42)	1.64	159	0.10	-	0.17
Informative communication ^c^	29.54 (2.64)	29.77 (2.54)	28.41 (2.89)	2.48	159	0.01	0.22	−0.03
Respect ^c^	16.78 (1,41)	16.79 (1.41)	16.74 (1.43)	0.17	159	0.87	-	0.02
Assertiveness ^c^	15.33 (2.71)	15.32 (2.63)	15.37 (3,14)	−0.09	159	0.93	-	0.05

Note: *Df* = degrees of freedom. ^a^ Effect size calculated by Cohen’s d. ^b^ Correlations calculated using Pearson’s *R*. ^c^ EHC-PS subscale; EHC-PS = Health Professionals Communication Skills Scale. SE-12 = Spanish version of the Self-Efficacy in Communication Skills scale.

**Table 4:** Pre-and post-intervention comparisons of the variables assessed in the sample.

	Pre- *M (SD)*	Post- *M (SD)*	*F*	*Df*	*p*	Effect Size ^b^
Attitudes towards health communication	52.54 (2.45)	53.11 (2.82)	0.67	1	0.42	-
SE-12	81.73 (14.08)	95.09 (9.49)	165.41	1	0.000	0.51
EHC-PS (total scale)	87.41 (8.08)	91.56 (6.94)	50.54	1	0.000	0.24
Empathy ^a^	25.76 (3.17)	27.35 (2.50)	55.40	1	0.000	0.26
Informative communication ^a^	29.54 (2.64)	30.82 (2.33)	1.20	1	0.28	-
Respect ^a^	16.78 (1.41)	17.21 (1.15)	12.58	1	0.001	0.07
Assertiveness ^a^	15.33 (2.71)	16.18 (3.31)	10.17	1	0.002	0.06

Note: *Df* = degrees of freedom. EHC-PS = Health Professionals Communication Skills Scale. SE-12 = Spanish version of the Self-Efficacy in Communication Skills scale. Sex was included as a co-variable for the ANOVA MLG in the attitudes variable and informative communication dimension. ^a^ EHC-PS subscale. ^b^ Eta squared.

## Data Availability

The data supporting the conclusions of this article will be available from the corresponding author upon request.
